# Focal Active Colitis Presented With Chronic Diarrhea

**DOI:** 10.7759/cureus.8140

**Published:** 2020-05-15

**Authors:** Bayarmaa Mandzhieva, John Taylor, Hammad Zafar, Mamoon Ur Rashid, Abu H Khan

**Affiliations:** 1 Internal Medicine, AdventHealth Orlando, Orlando, USA; 2 Internal Medicine, AdventHealth, Orlando, USA; 3 Internal Medicine, Advent Health, Orlando, USA; 4 Gastroenterology, AdventHealth, Orlando, USA

**Keywords:** chronic diarrhea, focal active colitis, cryptitis, xifaxan, irritable bowel syndrome, infectious colitis, inflammatory bowel disease.

## Abstract

There are various etiologies of colonic injury and inflammation. The most commonly described colitides in clinical practice are associated with infection, inflammatory bowel disease, ischemia, radiation and medications. The colonic wall has a limited set of responses to different types of injury; therefore, there is overlap between many of these disorders. Focal active colitis is characterized by isolated neutrophilic cryptitis with the background mucosa displaying normal crypt architecture. This inflammatory pattern can be easily unnoticed by pathologists because on low-power examination the mucosa may have almost normal appearance. General practitioners also may not be familiar with this term, underlying etiologies, associated risk factors, course, available therapies and follow up.

We present a case of an 82-year-old female with chronic diarrhea and weight loss. She had a negative infectious workup and normal radiology series. She subsequently underwent endoscopic evaluation in lieu of persistent and debilitating symptoms which revealed nonspecific macroscopic findings with pathology noting focal active colitis. She was empirically treated with a 14-day course of Xifaxan and responded well to management with almost complete resolution of her symptoms and no recurrence on six-month follow-up.

## Introduction

Focal active colitis (FAC) is a histologic term that describes the isolated finding of focal infiltration of the colonic crypts by neutrophils. Significant controversy is still present around the clinical implication of the diagnosis of FAC, which may or may not be clinically relevant.

Prior studies have shown that the incidence of Crohn’s disease (CD) in adults presenting with FAC was relatively low and has varied between 0% and 13%, whereas the incidence of infectious-type colitis has been demonstrated to be nearly 50% with FAC appearing to be an incidental finding without clinical relevance in about 25% of the patients [1,2]. In addition, based on clinical presentation and outcomes, a greater number of FAC cases in adults have been found to have an infectious etiology, although specific infectious pathogens were not detected in the majority of cases [3]. This can be partially explained by the fact that some of them are notoriously fastidious and may be difficult to recover. FAC is also encountered in patients with irritable bowel syndrome (IBS) and drug-induced colonic injury. The aim of this article is to present manifestations of FAC with chronic diarrhea and weight loss which is not a frequently encountered problem for general physicians. Our case also demonstrates effective treatment of FAC with Xifaxan, which is quite rarely reported upon extensive review of medical literature. Xifaxan could be one of the potential therapies for this condition, and our case emphasizes the necessity to conduct further research on underlying pathogenesis of FAC and its therapeutic options given the lack of strong recommendations and standardized approach available currently.

## Case presentation

An 82-year-old female presented to the gastroenterology office with complaints of diarrhea, nausea, abdominal cramping, increased flatulence and anorexia for the past three months. She also estimated that she had lost nearly 20 lbs in the past six months unintentionally.

She described the diarrhea as explosive and the amount of diarrhea correlating to the amount of food she was eating, but not the type of food. She reported taking a recent course of Flagyl for three days with no symptom improvement and no other recent exposure to antibiotics. She had tried Imodium without relief. She denied fever, chills, emesis, constipation, jaundice, melena, hematochezia, mucus in the stools, tenesmus, chest pain, respiratory or urinary symptoms.

She denied similar episodes in the past, any recent changes to her medications, chronic nonsteroidal anti-inflammatory drugs (NSAIDs) use, sick contacts or recent international travel. She had a history of giardia infection more than 10 years ago and had a normal colonoscopy in 2007.

Her past medical history was significant for intraductal carcinoma in situ of left breast, coronary artery disease, hypertension, hyperlipidemia, thyrotoxicosis, peripheral artery disease and osteopenia. Her past surgical history included left breast lumpectomy, hysterectomy, cholecystectomy, left renal mass removal and appendectomy. Family history was negative for inflammatory bowel disease (IBD) and gastrointestinal tract malignancies. She had a remote history of smoking and denied alcohol or illicit drug use.

In the office at the initial presentation, vital signs were within normal limits and physical examination was unremarkable. A complete blood count, comprehensive metabolic panel and thyroid-stimulating hormone were normal. She had negative stool studies for *Clostridium difficile* and negative stool cultures for *Vibrio*, *Yersinia*, *Shigella*, *Salmonella*, *Campylobacter *and *Escherichia coli *0157:H7.

**Figure 1:**
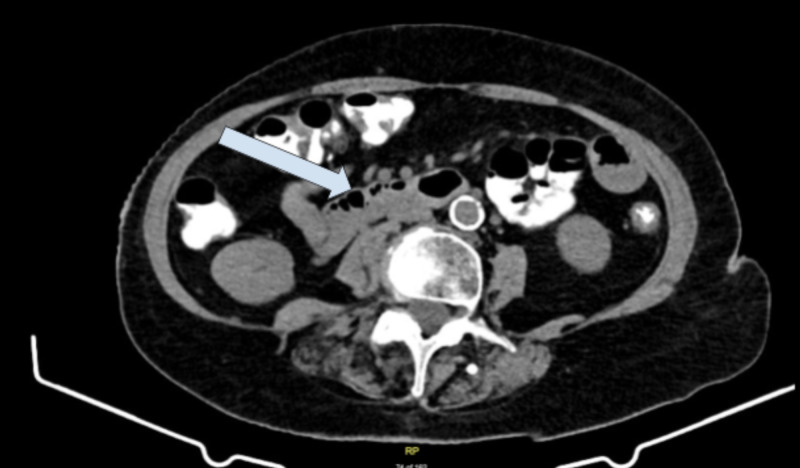
CT of the abdomen/pelvis with contrast No bowel wall thickening or obstruction. No mesenteric stranding or free fluid.

She underwent a CT of the abdomen and pelvis with contrast prior to her initial office visit, ordered by her urologist for evaluation of resolved flank pain, which was negative for acute intra-abdominal abnormalities (Figure 1).

She was recommended to complete stool studies with giardia antigen and ova and parasite microscopic exam, and she was scheduled for a diagnostic colonoscopy with biopsies. Stool studies returned negative for infectious etiologies. Given significant unintentional weight loss in an elderly patient, esophagogastroduodenoscopy (EGD) was ordered as well.

She was empirically treated with a trial of Xifaxan three times daily for 14 days for possible IBS with diarrhea (IBS-D) given previously failed treatment with Imodium and Flagyl. She was also given FDgard two tablets twice daily for symptomatic management of abdominal cramps.

She completed bowel preparation for colonoscopy with SUPREP® Bowel Prep Kit (Braintree Laboratories, Braintree, MA) (sodium sulfate, potassium sulfate and magnesium sulfate).

**Figure 2:**
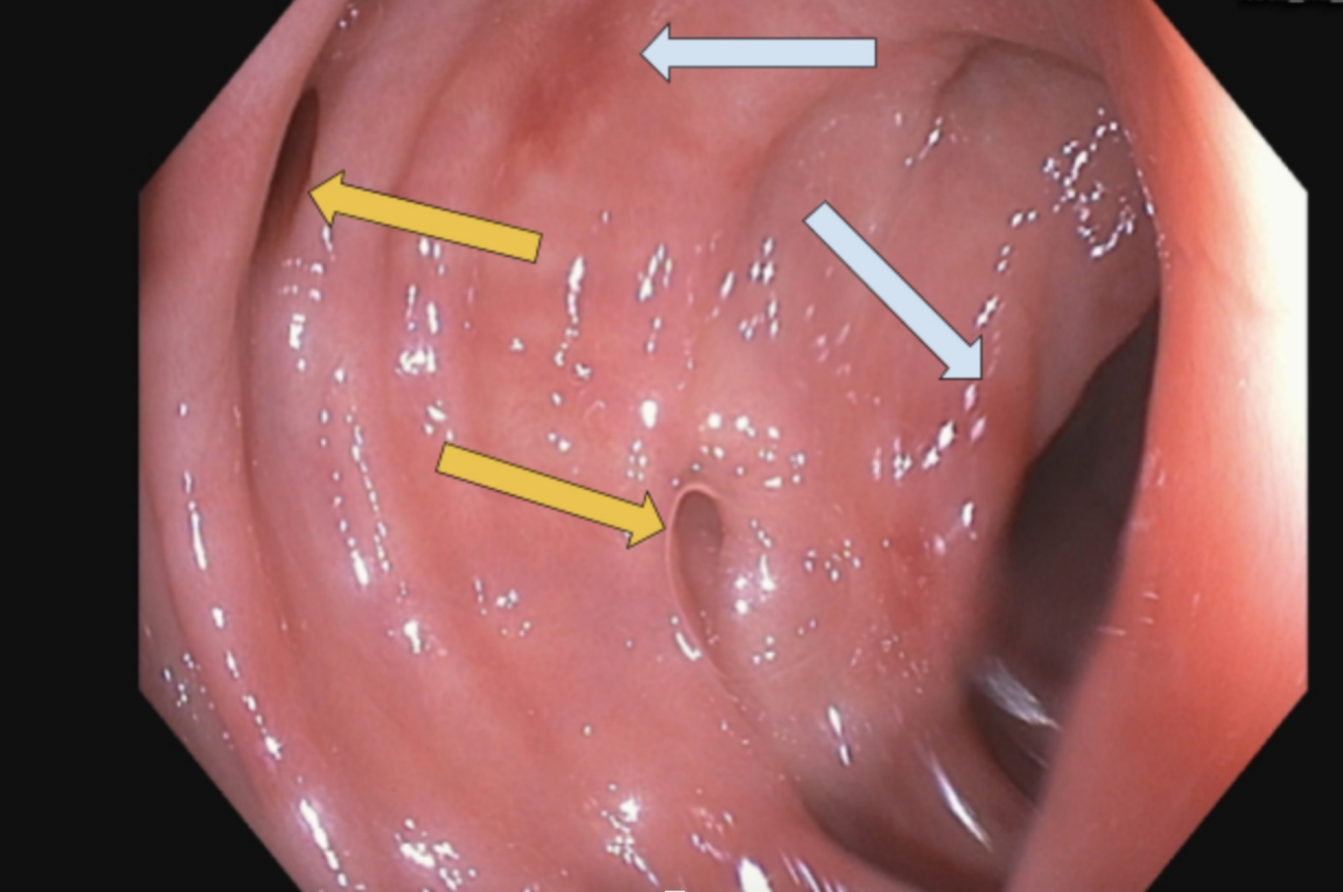
Colonoscopy with good bowel preparation Mild patchy mucosal erythema involving sigmoid colon (gray arrows). Few scattered diverticula (yellow arrows).

EGD and colonoscopy with good bowel preparation noted mild *Helicobacter pylor*i negative gastritis, patchy erythema in the sigmoid colon (Figure 2) with pathology noting FAC, 5-mm hyperplastic polyp in the sigmoid, diverticulosis and medium sized internal and external hemorrhoids. Small bowel biopsies ruled out celiac disease and multiple random biopsies from each segment of the colon were negative for microscopic colitis. A small 5-mm sessile polyp in the sigmoid colon was completely removed with cold snare. Two endoclips were deployed prophylactically at the polypectomy site.

Given colonoscopy findings, she was diagnosed with acute focal colitis and a decision was made to complete the course of Xifaxan and to consider switching to ciprofloxacin and Flagyl in the event of treatment failure. On the two-week follow-up appointment, she reported significant improvement in her symptoms (mainly diarrhea, abdominal cramps and flatulence) after the 14-day course of Xifaxan. The patient was advised to follow a high-fiber diet with over-the-counter Benefiber or Metamucil two to three times per day and low FODMAP (Fermentable Oligosaccharides, Disaccharides, Monosaccharides and Polyols) diet.

## Discussion

FAC pattern is characterized by a single focus or multiple foci of neutrophilic infiltration of the crypts with an otherwise unremarkable colonic mucosa. The background crypts are well preserved with no evidence of chronic injury [4].

Multiple etiologic entities can present as FAC on pathology reports. Most common clinical scenarios include acute self-limited infectious colitis, IBD early in its course, ischemic colitis, IBS, *Clostridium difficile* colitis, medication or chemical injury and bowel preparation artifact [4]. Medications associated with FAC are NSAIDs, mycophenolate acid, and ipilimumab, among others [5]. Although diarrhea is the most frequent reason for workup in the majority of these patients, FAC can also be an incidental finding as a result of Phospho-soda bowel preparation in asymptomatic patients undergoing screening colonoscopy [6]. Ozdil et al. found that 6.6% of IBS patients had FAC, and it may be eligible to take a routine biopsy in female patients and in patients aged above 50 years [7].

Osmond et al. in their retrospective review of cases of FAC in pediatric patients reported the following presenting clinical symptoms: diarrhea, rectal bleeding, abdominal pain, vomiting and others [8].

Of these patients, most presented as an infectious or acute self-limited colitis with a significant subset of the patients being immunocompromised. During the subsequent follow-up, none of the patients were later found to have either ulcerative colitis or CD [1].

Chronic diarrhea sometimes can be a very challenging clinical scenario due to multitude of differential diagnoses to consider. The majority of the macroscopic findings during endoscopic evaluation are unremarkable or nonspecific [9]. Greenson et al. in their study on adult population reported that endoscopic findings of FAC varied. Some of the patients had completely normal endoscopic findings, whereas others had scattered aphthous ulcers on normal background mucosa or patchy erythema [1]. Thus, the patient's past medical history, medication review and clinical correlation are crucial.

FAC is a histopathological diagnosis. The hallmark is neutrophilic cryptitis which involves several adjacent crypts on a single biopsy fragment. The lamina propria is often hypercellular with normal architecture of the background mucosa and no Paneth cell metaplasia. The presence of apoptotic crypt eplithelial cells is variable^ ^[10].

Features on histology cannot distinguish between incidental and clinically significant FAC. Hence, it remains a diagnostic challenge in which features on pathology must appropriately fit the correct clinical presentation.

Management of FAC itself is not well described. Therefore, physicians generally use their clinical judgment and treatment available for presumed underlying etiology, such as IBD, IBS or acute infectious colitis.

The clinicopathological features of CD and certain enteric pathogens can mimic each other to some degree and can all present with an endoscopic picture of focal/segmental enterocolitis. Most notable microorganisms in this regard are *Campylobacter*, *Salmonella*, *Shigella*, *Yersinia *infections, *Escherichia coli* serotype O157:H7, *Chlamydia trachomatis,* *Mycobacterium tuberculosis*, *Actinomyces *spp, *Entamoeba histolytica,* *Giardia lamblia* and others [11]. Normal gut flora might become pathogenic under certain circumstances as well.

There is evidence that supports the notion that bacteria act as the driving force for both active and chronic intestinal inflammation. One hypothesis is that chronic inflammation may be due to abnormal immune responses to the normal gut flora. These notions support the use of antibiotics in the case of active intestinal inflammation and also brings up the idea of manipulating the enteric flora as a possible therapeutic for the more chronic cases [12].

IBS has been attributed to many triggers with numerous studies suggesting that up to one-third of IBS-D cases result from a prior bacterial infection [13].

The use of oral antibiotics has likely therapeutic utility in multiple enteric conditions with these notions in mind. However, several barriers exist to antibiotic use such as concerns for growing bacteria resistance, interactions with other drugs and antibiotic side effects. The use of minimally absorbed antibiotics, which are more gut targeted, may provide a solution to these concerns [14].

Rifaximin (Xifaxan) is a safe, rifamycin-derived antibiotic with a wide range of antimicrobial spectrum covering most Gram-positive and Gram-negative bacteria, including aerobes and anaerobes . It has very poor systemic absorption after oral administration and complete fecal excretion as unchanged drug [12].

Rifaximin has several indications, which include select cases of infectious diarrhea, hepatic encephalopathy and surgical prophylaxis. The drug has also found use in the prevention of small intestinal bacterial overgrowth, *Clostridium difficile* associated colitis, diverticular disease and infectious diarrhea. It may also have benefit in other conditions such as IBD and IBS [14].

Different studies suggested that it could be beneficial in the treatment of active IBD refractory to the standard treatment. For example, Shafran et al. presented recently an open-label study on the efficacy and safety of rifaximin 600 mg/day for 16 weeks in the treatment of mildly to moderately active CD. At the end of the study, 59% of patients were in remission [15].

Rifaximin proved more effective than placebo for global symptoms and bloating in IBS patients. The modest therapeutic gain was similar to that yielded by other currently available therapies for IBS [13].

Banaag et al. in their multicenter retrospective study have shown that there are approximately 25% of patients presenting with diarrhea, abdominal pain and IBS with the histological finding of FAC without a definitive diagnosis. More than 50% of patients who had diarrhoea had FAC, and of these 44% responded to 5-aminosalicylic acid (5ASA), evident by resolution of symptoms within two to six weeks [16].

In summary, FAC remains a diagnostic challenge. One must incorporate the patient's presenting history, medications and findings on endoscopy to help narrow the differential diagnosis. Histology can be helpful; however, there is no specific finding or feature that can distinguish between incidental and clinically significant FAC (such as a minimum neutrophil count).

As such, further studies should be conducted to determine patterns of histological disease and/or a threshold of inflammation so that one can predict disease trajectory and allow for better patient stratification. 

We presented the case of FAC being effectively treated with rifaximin.

Rifaximin is a very promising agent with an excellent safety profile and more experimental studies with rifaximin in clinical practice will help to further define the role of this antibiotic in gastroenterology.

## Conclusions

Our case is unique because infectious pathogens were not identified and she did not have risk factors commonly associated with FAC in the literature such as IBD, NSAIDs use or bowel preparation with Phospho-soda. She achieved significant resolution of her diarrhea within two weeks of Xifaxan treatment with no recurrence for at least six months. In summary, FAC should be strongly considered in the differential diagnosis in patients presenting with diarrhea and macroscopically normal mucosa. The case also highlights the necessity of gaining more experience in treatment of FAC with Xifaxan.

## References

[1] Greenson JK, Stern RA, Carpenter SL, Barnett JL (1997). The clinical significance of focal active colitis. Hum Pathol.

[2] Volk EE, Shapiro BD, Easley KA, Goldblum JR (1998). The clinical significance of a biopsy-based diagnosis of focal active colitis: a clinicopathologic study of 31 cases. Mod Pathol.

[3] Schneider EN, Havens JM, Scott MA (2006). Molecular diagnosis of Campylobacter jejuni infection in cases of focal active colitis. Am J Surg Pathol.

[4] Assarzadegan N, Montgomery E, Pezhouh MK (2017). Colitides: diagnostic challenges and a pattern based approach to differential diagnosis. Diagn Histopathol.

[5] Marginean EC (2016). The ever-changing landscape of drug-induced injury of the lower gastrointestinal tract. Arch Pathol Lab Med.

[6] Driman DK, Preiksaitis HG (1998). Colorectal inflammation and increased cell proliferation associated with oral sodium phosphate bowel preparation solution. Hum Pathol.

[7] Ozdil K, Sahin A, Calhan T (2011). The frequency of microscopic and focal active colitis in patients with irritable bowel syndrome. BMC Gastroenterol.

[8] Osmond A, Ashok D, Francoeur CA, Miller M, Walsh JC (2018). Is focal active colitis of greater clinical significance in pediatric patients? A retrospective review of 68 cases with clinical correlation. Hum Pathol.

[9] Lin W-C, Chang C-W, Chen M-J (2017). Challenges in the diagnosis of ulcerative colitis with concomitant bacterial infections and chronic infectious colitis. PLoS One.

[10] Jessurun J (2017). The differential diagnosis of acute colitis: clues to a specific diagnosis. Surg Pathol Clin.

[11] Hertogh GD, Aerssens J, Geboes KP, Geboes K (2008). Evidence for the involvement of infectious agents in the pathogenesis of Crohn’s disease. World J Gastroenterol.

[12] Gionchetti P, Rizzello F, Morselli C, Romagnoli R, Campieri M (2005). Management of inflammatory bowel disease: does rifaximin offer any promise?. Chemotherapy.

[13] Menees SB, Maneerattannaporn M, Kim HM, Chey WD (2012). The efficacy and safety of rifaximin for the irritable bowel syndrome: a systematic review and meta-analysis. Am J Gastroenterol.

[14] Su CG, Aberra F, Lichtenstein GR (2006). Utility of the nonabsorbed (<0.4%) antibiotic rifaximin in gastroenterology and hepatology. Gastroenterol Hepatol.

[15] Shafran I, Johnson LK (2005). An open-label evaluation of rifaximin in the treatment of active Crohn’s disease. Curr Med Res Opin.

[16] Banaag MJM, Daulat EE, Mediodia L (2013). Mo1338 is focal active colitis a new miscellany of inflammatory bowel disease? And is there a role for 5 ASA in the management of FAC?. Gastroenterology.

